# Neurogenin2 regulates the initial axon guidance of cortical pyramidal neurons projecting medially to the corpus callosum

**DOI:** 10.1186/1749-8104-6-30

**Published:** 2011-08-24

**Authors:** Randal Hand, Franck Polleux

**Affiliations:** 1 Neuroscience Center, Department of Pharmacology, School of Medicine, University of North Carolina at Chapel Hill, Chapel Hill, NC 27599, USA; 2 Solomon H Snyder Department of Neuroscience - The Johns Hopkins School of Medicine, 725 N. Wolfe Street, 1001 PCTB, Baltimore, MD 21205, USA; 3 Dorris Neuroscience Center, Department of Cell Biology, The Scripps Research Institute, 10550 North Torrey Pines Road, DNC 202, La Jolla, CA, USA

## Abstract

**Background:**

The formation of the mammalian central nervous system requires the establishment of complex neural circuits between a diverse array of neuronal subtypes. Here we report that the proneural transcription factor Neurogenin2 (Ngn2) is crucial for the proper specification of cortical axon projections.

**Results:**

The genetic loss of Ngn2 in mice results in fewer callosal axons projecting towards the midline as well as abnormal midline crossing. shRNA-mediated knockdown of Ngn2 revealed its cell-autonomous requirement for the proper projection of axons from layer 2/3 pyramidal neurons to the midline *in vivo*. We found that the acute loss of Ngn2 *in vivo *induces the axon of superficial layer 2/3 neurons to project laterally towards aberrant cortical and subcortical targets.

**Conclusions:**

These and previous results demonstrate that Ngn2 is required for the coordinated specification of cardinal features defining the phenotype of cortical pyramidal neurons, including their migration properties, dendritic morphology and axonal projection.

## Background

The mammalian nervous system consists of a tremendous diversity of neuronal subtypes forming complex functional circuits. In the cerebral cortex, long-distance-projecting glutamatergic pyramidal neurons arise from radial glial progenitors located in the dorsal telencephalon [1]. During cortical neurogenesis in rodents, radial glial progenitors divide asymmetrically to generate another radial glial progenitor and an intermediate progenitor cell (IPC) that translocates to the subventricular zone (SVZ) [1]. These IPCs display a transient multipolar morphology characterized by the dynamic extension and retraction of immature neurites, which might sense their micro-environment and respond to cues polarizing their leading process (future apical dendrite) dorsally towards the cortical plate and their trailing process (future axon) ventrally [2,3]. During this polarization, the neuron adheres to a radial glial cell process and initiates radial migration through the cell-sparse but axon-rich intermediate zone (IZ) towards the pial surface. Upon reaching the top of the cortical plate, just below the pial surface, pyramidal neurons detach from the radial glial cell and undergo terminal translocation before elaborating both their dendritic and axonal processes. In mice, neurogenesis occurs between embryonic day 11 (E11) and E18 [4-6], giving rise to neurons accumulating in an 'inside-first outside-last' pattern where late-born neurons migrate to layers located more superficially than their predecessors [7]. Ultimately, the location of progenitors, mode of migration, neurotransmitter expression, dendritic morphology, and axonal projections are used to define subtypes of cortical neurons [8].

One of the defining features of pyramidal neurons is the type of axonal projections they form within the brain and spinal cord. Pyramidal neurons in the deep layers of the cortex (layers 5/6) project laterally to exit the cortex towards subcortical regions [9,10]. On the contrary, pyramidal neurons in superficial layers 2/3 mostly project to other cortical areas, including callosal projections through the midline that form the corpus callosum [9,10]. This initial choice to project medially or laterally is one of the earliest and most critical axon guidance decisions made by pyramidal neurons since it defines these two large and distinct classes of pyramidal projection neurons. However, the molecular mechanisms underlying the specification of this key axon guidance choice is still poorly understood [9].

Neurogenin2 (Ngn2) is a proneural basic helix-loop-helix (bHLH) transcription factor was first identified for its ability to promote neuronal differentiation in the brain and spinal cord [11,12]. Beyond its proneural function, Ngn2 also specifies the cardinal phenotypic features defining pyramidal neurons as a subpopulation, such as glutamatergic neurotransmitter expression, radial migration properties and their pyramidal dendritic morphology [13-17]. Here we show that Ngn2 also specifies the axon guidance choice made by superficial pyramidal neurons to project towards the midline *in vivo*.

## Materials and methods

### Animals

In this study we used several inbred strains of mice, including Balb/c and C57Bl/6 (for *in utero *electroporation). The Ngn2 green fluorescent protein (GFP) knockin mice were a generous gift from Dr Francois Guillemot and were maintained on a Balb/c background. Males and females were used indistinguishably for quantifications. All experiments were performed in strict accordance to IACUC protocols approved by UNC Chapel Hill.

### Plasmids

For this study, we created the pSCV2 construct from the pSilencer2.1 (Ambion (Austin, TX- USA)) vector. To achieve this, we inserted a CAG-Venus-pA cassette into the backbone of the pSilencer2.1 vector. All short hairpin RNA (shRNAs) are inserted downstream of the U6 promoter using BamHI and HindIII restriction sites. The targeting sequence of control shRNA is ACTACCGTTGTTATAGGTG. The target sequence of the shRNA targeting Ngn2 is CCAACAACCGCGAGCGCAA. To create the rescue mutant of Ngn2, a noncoding point mutation was generated by mutating nucleotide 360 from cytosine to adenine using the QuickChangeII mutagenesis kit from Stratagene (Santa Clara, CA - USA).

### Antibodies and immunostaining

All immunofluorescent staining was performed as previously described [15]. Primary antibodies were: anti-L1 (1:2,500; Millipore(Millipore (Billerica, MA-USA)), anti-GFP (1:2,000; Aves (Aves (Tigard, OR- USA)), anti-NF165 (1:1,000; Developmental Studies Hybridoma Bank at the University of Iowa), anti-CTIP2 (1:2,000; Abcam (Cambridge, MA- USA)), anti-Tbr1 (1:1,000; Chemicon (Billerica, MA- USA)), and anti-Cux1 (1:500; Santa Cruz Biotechnology (Santa Cruz, CA- USA)). Secondary antibodies were: goat anti-chicken Alexa 488, goat anti-rat Alexa 546, goat anti-rat Alexa 637, goat anti-rabbit Alexa 546, goat anti-rabbit Alex 637, and goat anti-mouse Alexa 546. Streptavidin conjugated to Alexa 546 was used to detect the biotin-labeled dextrose amines.

### Anterograde axonal tracings

Briefly, E18.5 brains dissected and injected with 0.1 mg/ml 10,000MW BDA Molecular Probes (Carlsbad, CA - USA) in phosphate-buffered saline (PBS) with 0.1% fast green for visualization. Then the brains were incubated in artificial cerebrospinal fluid at 37°C and oxygenated with 95/5% O_2_/CO_2 _for 8 hours. After 8 hours, brains were fixed overnight at 4°C in 4% paraformaldehyde in PBS pH7.4. The brains were then sectioned and immunostained.

### *Ex vivo *electroporation and organotypic slice culture

All *ex vivo *electroporation and organotypic slice cultures were performed essentially as described previously [15].

### *In utero *electroporation

Briefly, E15.5 mice were deeply anesthetized using 2.5% 2,2,2 tribromoethanol in PBS. A small 1- to 2-cm incision was made along the midline and the uterine horns were removed from the abdominal cavity and placed on sterile gauze. The lateral ventricles of the embryos were injected with 2 μg/μl of plasmid in 1 × PBS, and 0.1% fast green dye (for visualization). The embryos were subsequently electroporated as follows: 4 pulses of 30V for 50 ms with a 500-ms interval. After the embryos had been injected and electroporated, the uterine horns were placed back in the abdominal cavity and the incision was sutured. Mice were allowed to recover and embryos were harvested at postnatal day 14. All surgeries strictly adhered to IACUC approved protocols.

## Results

**Figure 1:**
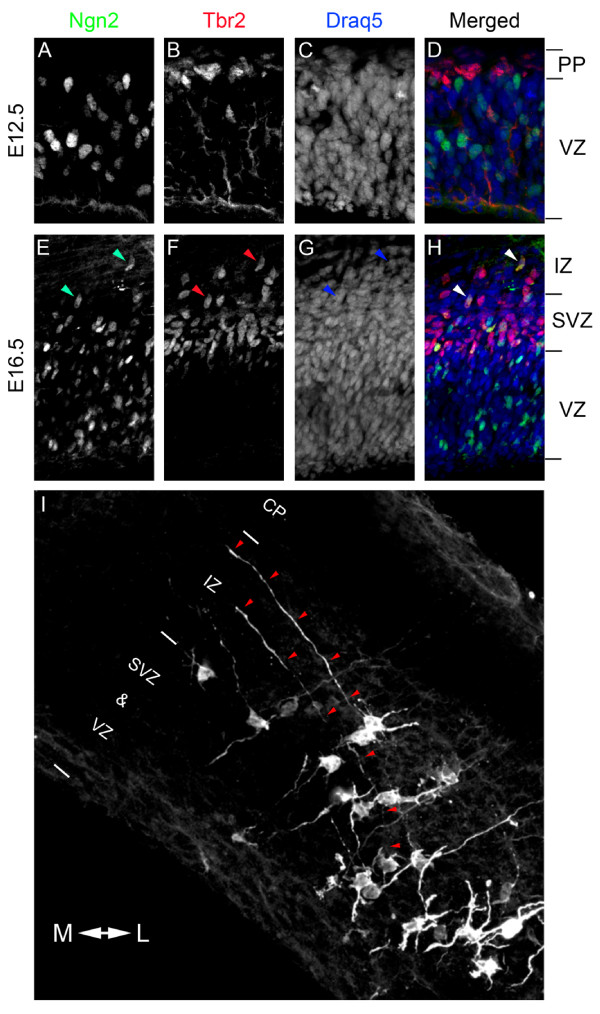
**Pattern of Ngn2 expression in the developing cortex**. **(A-H) **Coronal sections from E12.5 (A-D) and E16.5 (E-H) were immunostained for Ngn2 (A,E), Tbr2 (B,F) and Draq5 (C,G). (D,H) Merged images. At E12.5, Ngn2 expression is resticted to the VZ (A) and has little colocalization with Tbr2 (B,D), a marker for intermediate progenitors. In contrast, at E16.5, a time when superficial layer neurons are generated, Ngn2 is expressed widely in the VZ and SVZ, and occasionally in the IZ (E); many cells also co-stain for Ngn2 and Tbr2 (F,H), demonstrating that many intermediate progenitors express Ngn2. Arrowheads denote cells co-expressing Ngn2 and Tbr2 located in the upper SVZ and IZ. **(I) **To determine when callosal axons first emerge, E15.5 cortices were electroporated with the YFP variant Venus and cultured as organotypic slices. Electroporated neurons were immunostained for GFP 36 hours after electroporation to visualize axon initiation. At this time point, several neurons have a single long neurite growing medially (red arrowheads). Interestingly, none of these neurons have formed a leading process, which is characteristic of radially migrating neurons. CP, cortical plate; IZ, intermediate zone; PP, preplate; SVZ, subventricular zone; VZ, ventricular zone.

Temporal pattern of Ngn2 expression relative to axogenesis of callosally projecting neurons Ngn2 is expressed within the germinal zones of the cerebral cortex during development [15,18]. At early stages of neurogenesis (E10 to E12), Ngn2 is primarily expressed in actively dividing neural progenitor cells (NPCs) located in the ventricular zone (VZ) [18,19] and not within the preplate, where newly differentiated neurons are located (Figure 1A-D). Interestingly, as neurogenesis progresses, Ngn2 expression shifts from being expressed primarily in actively dividing NPCs at early time points (E10 to E12) to being expressed primarily in newly differentiated neurons at later time points (after E14.5) [18,19]. Our expression data confirm this shift as Ngn2 expression is found in the VZ, SVZ, and occasionally in the IZ at E16.5 (Figure 1E-H). Additionally, at E16.5 many of the Ngn2-positive cells also express the transcription factor Tbr2 (Figure 1E-H). Tbr2 is expressed in IPCs [20] and is necessary for their production [21]. We observe frequent co-expression of Ngn2 and Tbr2 in the SVZ/IZ. This temporal pattern of expression suggests that Ngn2 may regulate the transition from a multipolar IPC to a highly polarized migrating neuron with a unipolar leading process (future apical dendrite) and a trailing process (future axon).

To visualize if late Ngn2 expression in the SVZ/IZ matches temporally with axon initiation of callosally projecting neurons that are generated between E15.5 and E18.5 [6], we used *ex vivo *electroporation coupled with organotypic slice culture. This technique effectively introduces cDNA into NPCs, and within 24 hours post-electroporation, neurons begin to differentiate from the NPCs [15]. To ensure we labeled superficial layer neurons, we electroporated cortices at E15.5, a time point when only superficial neurons would be labeled [6,22]. To visualize the emerging axons, we optimized electroporation conditions to allow for single cell resolution using a plasmid encoding the yellow fluorescent protein (YFP) variant Venus and imaged cells 36 hours post-electroporation. At this time point, we found many newly differentiated neurons containing a long single neurite (Figure 1I). Interestingly, these single long neurites grew medially towards the corpus callosum within the IZ, strongly suggesting that these are presumptive axons. In these neurons, the emergence of axons appears to precede the formation of a leading process and the initiation of radial migration. This refines previously published data [23,24] and shows that the directed emergence of the axon occurs very soon after cell cycle exit, correlating well with Ngn2 expression. Importantly, these results strongly suggest that the decision for a neuron to project an axon medially and to become a callosally projecting neuron is taken extremely early during neuronal differentiation, well before the neurons reach their final position in the cortical plate.

### Ngn2 is required for callosal axon projection and midline crossing

**Figure 2:**
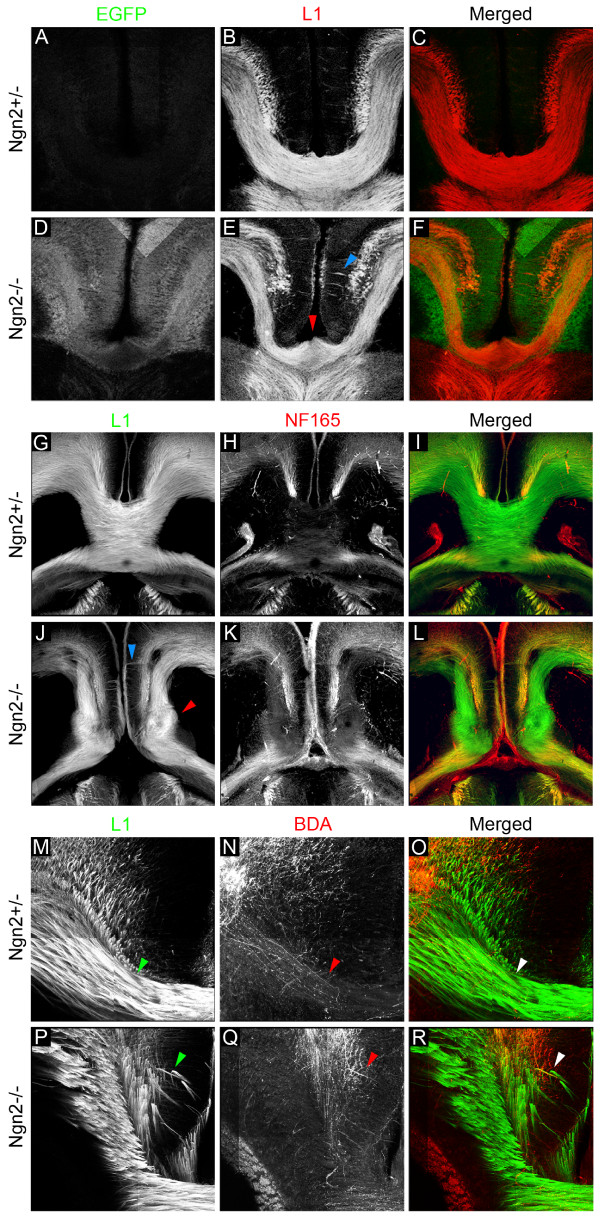
**Ngn2 regulates callosal axon projection and corpus callosum formation *in vivo***. **(A-F) **Coronal sections of E18.5 Ngn2+/- embryos (A-C) and Ngn2-/- (D-F) embryos were immunostained with EGFP (A,D) since EGFP was inserted into the Ngn2 locus to create the null allele. The sections were also immunostained with the axonal marker L1 (B,E) to reveal many of the cortical axon tracts. Merged EGFP and L1 images (C,F) show a reduction in callosal axons in Ngn2-/- embryos compared to the heterozygous embryos. The immunostaining also revealed malformations of the corpus callosum, including aberrant axonal projections at the midline and an abnormal corpus callosum (blue arrowhead, E). **(G-L) **Horizontal sections from E18.5 Ngn2+/- embryos (G-I) and Ngn2-/- (J-L) embryos were immunostained for the axonal markers L1 (G,J) and NF165 (H,K). (I,L) Merged images. The immunostaining of axonal markers shows a reduction of callosal axons and the lack of a corpus callosum (J-L), the formation of probst bundles (red arrowhead, J), and misprojecting axons at the midline (blue arrowhead, J) in the embryos lacking Ngn2 None of these phenotypes were observed in the heterzygous embryos. **(M-R) **Ngn2+/- cortices (M-O) and Ngn2-/- (P-R) cortices were injected with the anterograde tracer BDA at E18.5 to determine if the axons that prematurely defasciculate orginated from cortical neurons. Coronal sections were immunostained with the axonal marker L1 to reveal callosal axons (M,P). The BDA tracings were revealed with streptavidin conjugated with Alexa 546 (N,Q). (O,R) Merged images. Arrowheads in M-O indicates callosal axons appropriately reaching the midline. Prematurely defasciculating axons were revealed in the Ngn2-/- embryos by both L1 staining and BDA anterograde tracings (P-R, arrowheads). This phenotype was not observed in Ngn2+/- embryos (M-O). BDA anterograde tracings definitively demonstrate that these axons are cortical in orgin.

To assess the role for Ngn2 in regulating the axonal projection of pyramidal neurons, we began by assessing cortical axon projections in the Ngn2 knockin mouse. The Ngn2 knockin mouse has a gene encoding enhanced GFP (EGFP) inserted into the Ngn2 coding sequence, creating a null allele that faithfully reports the expression of Ngn2 [25] (from this point forward the Ngn2 homozygous knockin mouse will be referred to as the Ngn2-/- mouse). We began our study by harvesting Ngn2+/- and Ngn2-/- embryos at E18.5 since the Ngn2-/- mice are perinatally lethal [26]. We immunostained coronal sections from E18.5 embryos with the axonal marker L1, which labels most of the cortical axon tracts and allowed us to assess major tract formation in the embryonic brains (Figure 2A-L). Interestingly, we found a dramatic decrease in the number of callosal axons in many Ngn2-/- embryos (Figure 2D-F, J-L) compared to the Ngn2+/- embryos (Figure 2A-C, F-I). Careful examination of these embryos also revealed abnormalities at the midline. Often the Ngn2-/- embryos had an abnormal corpus callosum with a reduced thickness (46% of embryos, N = 13; Figure 2D-F), and less frequently, the Ngn2-/- embryos completely lacked a corpus callosum (15% of embryos, N = 13; Figure 2J-L). Interestingly, the embryos containing a malformed corpus callosum often had axons appearing to defasciculate prior to reaching the midline (arrowheads in Figure 2E, J). To determine if the defasciculating axons originated from ipsilateral cortical neurons, we used biocytin (or biotinylated dextran amine; BDA) to anterogradely trace cortical axons. Ngn2+/- and Ngn2-/- cortices were injected with BDA at E18.5 to label cortical projecting axons. The brains were sectioned and counterstained with L1 to visualize callosal axons and the BDA anterograde tracings were visualized using Alexafluor-conjugated streptavidin (arrowheads in Figure 2M-R). As suspected, the BDA anterograde tracings revealed axons from cortical neurons in the Ngn2-/- embryos defasciculating prior to reaching the midline (arrowheads in Figure 2P-Q). This premature axon defasciculation phenotype was never found in Ngn2+/- embryos (Figure 2M-O). In total we examined ten litters from Ngn2+/- heterozygous matings. We found no defect in any axonal tracts from the 12 Ngn2+/- embryos we examined. In the 13 Ngn2-/- embryos examined, we found 46% had a significant decrease in thickness of the corpus callosum, suggesting a reduced number of callosal axons, 31% had malformations and hypoplasia of the corpus callosum, and 15% had a complete agenesis of the corpus callosum. Our data demonstrate that Ngn2 regulates the number of callosal axons reaching the midline, as well as the guidance of callosal axons at the midline, and the formation of the corpus callosum.

### Ngn2 regulates the initial guidance of callosal axons

**Figure 3:**
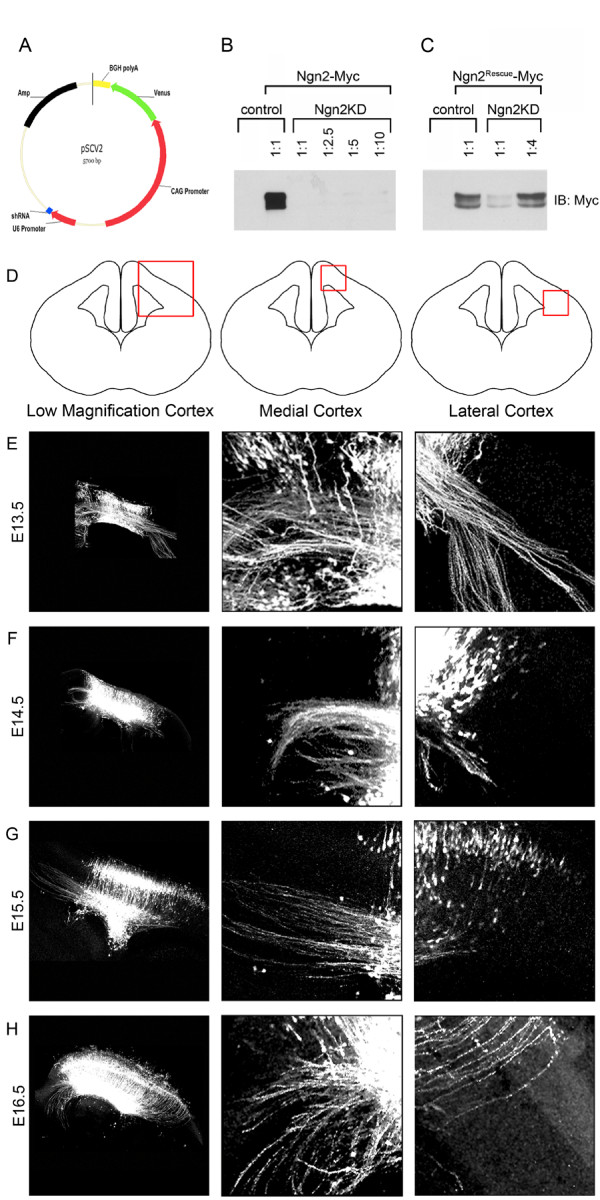
**shRNA-mediated knockdown of Ngn2 and time course of axonal projection using *ex vivo *electroporation coupled with organotypic slice culture**. **(A) **The bicistronic pSCV2 plasmid was engineered to allow for the visualization of cells expressing the shRNA. This plasmid contains a nonspecific control shRNA under the control of the pol-III U6 promoter and the YFP variant Venus under control of the pol-II CAG promoter. **(B,C) **The control vector with the nonspecific shRNA does not reduce expression of Myc-tagged Ngn2 in P19 cells. (B) Myc-tagged Ngn2 expression is drastically reduced in the presence of shRNA targeting Ngn2 (Ngn2KD). This shRNA effectively knocks down Ngn2 expression even at a 1:10 ratio of shRNA to Myc-tagged Ngn2 plasmids. (C) In order to rescue shRNA-mediated knockdown of Ngn2, we generated a Ngn2 rescue mutant (Ngn2^Rescue^) containing a single noncoding point mutation that reduces the ability of the shRNA to target Ngn2. A 1:1 ratio of shRNA to myc-tagged Ngn2^Rescue ^allows for weak expression of Ngn2, and a 1:4 ratio of shRNA to myc-tagged Ngn2^Rescue ^allows for expression of Ngn2 equal to control shRNA. **(D) **Diagrams depicting the areas imaged in (E-H): left, low magnification images reveal the area of the cortex electroporated; middle, high magnification of the medial cortex to visualize medial projecting axons; right, high magnification of the lateral cortex to visualize lateral projecting axons. **(E-H) **For the axonal projection time course, embryonic cortices were electroporated with a plasmid encoding the YFP variant Venus under the control of the CAG promoter at time points varying from E13.5 to E16.5. After 5 days *in vitro *(DIV), the electroporated neurons were immunostained using anti-GFP to assess the axonal projections of cortical neurons labeled at each time point. (E) At E13.5, a large proportion of the electroporated neurons have laterally projecting axons. Starting at E14.5, the number of laterally projecting axons steadily decreases (F), and by E15.5 there are very few laterally projecting axons (G). (H) Virtually no laterally projecting axons were seen following electroporation at E16.5.

The reduction of the size of the corpus callosum could be due to a reduction in the number of neurons specified to project medially. While it is clear that Ngn2 regulates callosal axon projections, it is unclear whether these effects are cell-autonomous or non-autonomous. We tested the cell autonomy of Ngn2 effects on callosal axons by documenting the emergence of callosal axons from newly differentiated neurons. We generated a bicistronic plasmid that allows simultaneous expression of a shRNA under the RNA polymerase III-specific U6 promoter and the YFP variant Venus under the RNA polymerase II-specific chicken β-actin (CAG) promoter (Figure 3A). We generated a control plasmid (pSCV2) that expresses a nonspecific shRNA and plasmids containing shRNAs targeting Ngn2. We tested the effectiveness of the shRNA-mediated knockdown of Ngn2 in P19 cells. We found that the shRNA targeting Ngn2 effectively knocks down myc-tagged Ngn2 even when transfected at a 1:10 ratio of shRNA relative to myc-tagged Ngn2 (Figure 3B). We also generated a 'rescue' mutant of Ngn2 (Ngn2^Rescue^) in which a single noncoding point mutation was created within the seed region targeted by the shRNA. This noncoding point mutation strongly reduced the ability of the shRNA to target Ngn2 (Figure 3C) and allowed us to control for potential off-target effects of our Ngn2-targeted shRNA.

*Ex utero *cortical electroporation coupled with organotypic slice culture is ideal for rapidly assessing the many aspects of pyramidal neuron differentiation and migration [15,23], so we began by performing a developmental time course to assess if the axonal projections of cortical neurons are maintained in slice cultures *in vitro*. Embryonic cortices were electroporated at times ranging from E13.5 to E16.5 and organotypic slice cultures were prepared and cultured for 5 days *in vitro *(DIV), allowing for generation, migration and axon projection of electroporated pyramidal neurons (Figure 3E-H). We found that the axonal projections of pyramidal neurons cultured *in vitro *mimicked those found *in vivo*. At the earliest time points (E13.5 and E14.5), we found a significant proportion of axons were projecting laterally as we would expect since deep layer neurons are generated at these developmental time points (Figure 3E, F). At later time points (E15.5 to 16.5), the axons of electroporated neurons almost exclusively projected medially (Figure 3G, H) as only superficial neurons would be generated at this later time point [6].

**Figure 4:**
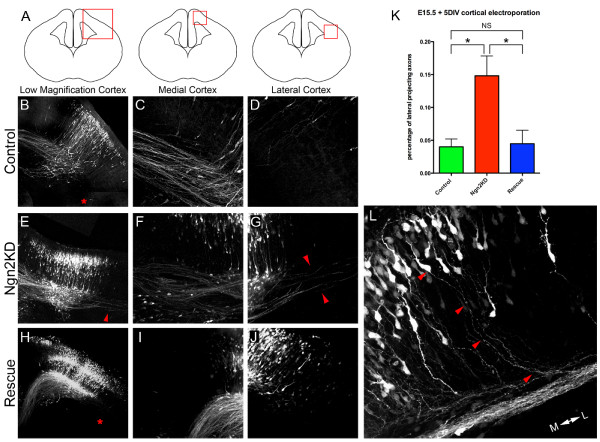
**Ngn2 is required cell autonomously for proper axon guidance of upper layer cortical neurons to the midline**. **(A-J) **To target upper-layer cortical neurons, the dorsal telencephalon of E15.5 wild-type embryos was electroporated with plasmids encoding the fluorescent protein Venus and either a control shRNA (B-D), shRNA targeting Ngn2 (E-G), or shRNA targeting Ngn2 plus Ngn2^Rescue ^mutant, which has a single non-coding point mutation that reduces the ability of the shRNA to target Ngn2 (H-J). Following electroporation, organotypic slice cultures were prepared and cultured for 5 DIV. (A) Diagrams depicting the areas that were imaged for the expression of Venus revealing the neurons and their axons containing the shRNA. (C,F,I) Regardless of the shRNA electroporated, all conditions contain medial (callosal) projecting axons. Interestingly, the neurons containing only the shRNA targeting Ngn2 had a dramatic increase in the number of lateral projecting axons (red arrowheads, G), and many of these neurons were located within the cortical plate (L). **(K) **Quantification of the axonal projections demonstrates a significant increase in the number of lateral projecting axons in neurons containing only the shRNA targeting Ngn2. NS, not significant; *P < 0.05 according to ANOVA test.

To test the cell autonomous regulation of axon projection by Ngn2, E15.5 cortices were electroporated with either control shRNA (control), shRNA targeting Ngn2 (Ngn2KD), or shRNA targeting Ngn2 plus the Ngn2^Rescue ^mutant (Rescue) and cultured for 5 DIV. As expected, the majority of pyramidal neurons expressing control shRNA displayed medially projecting axons (Figure 4B-D, K). Interestingly, neurons containing the shRNA targeting Ngn2 had a significant increase in laterally projecting axons (*P *< 0.01; Figure 4E-G, K). High magnification of the lateral cortex reveals the presence of neurons within the cortical plate with lateral projections (arrowheads in Figure 4L). To confirm that the presence of axons erroneously projecting laterally is a Ngn2-specific effect, we electroporated cortices with both the shRNA targeting Ngn2 and the Ngn2^Rescue ^mutant. As expected, the neurons containing both the shRNA targeting Ngn2 and the Ngn2^Rescue ^mutant had significantly fewer laterally projecting axons when compared to the neurons containing the shRNA targeting Ngn2 alone (*P *< 0.05; Figure 4H-J, K) and no significant increase in laterally projecting axons compared to the control shRNA (Figure 4H-J, K). Based on the relatively low transfection efficiency achieved using *ex utero *cortical electroporation, our data from the acute knockdown of Ngn2 expression suggest that Ngn2 regulates the axon guidance of callosal axons in a cell-autonomous manner. These data combined with the data from the Ngn2-/- embryo demonstrate that Ngn2 is required in layer 2/3 neurons for the guidance of their axon towards the midline. Furthermore, our evidence suggests that the initial axon guidance of pyramidal neurons is an early event occurring just after neuronal differentiation and well before completion of neuronal migration to their final destination in the cortical plate.

### Loss of Ngn2 does not alter the laminar fate of cortical neurons

**Figure 5:**
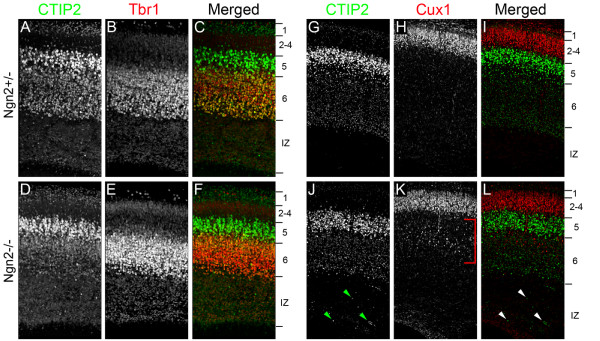
**Ngn2-/- embryos have normal cortical lamination**. **(A-L) **To assess any changes in cortical lamination upon the loss of Ngn2, we harvested Ngn2+/- embryos and Ngn2-/- embryos at E18.5 (A-F), and at the last possible time point, P0 (G-L). To assess deep layer formation, we immunostained sections with CTIP2, which strongly labels layer 5 and weakly labels layer 6a (A,D,G,J), and Tbr1 (B,E), which labels layer 6a. Merged images (C,F) revealed no difference in size or location of layers V and VI between Ngn2+/- (A-C) and Ngn2-/- embryos (D-F). To compare any differences in superficial layer formation, we immunostained sections with Cux1 (H,K), which labels layers 2 to 4. Again we observed no obvious difference in laminar formation when Ngn2+/- (G-I) and Ngn2-/- sections were stained for superficial layer markers. (I,L) Merged images. Immunostaining for laminar markers did reveal the presence of heterotopias in the intermediate zon of the Ngn2 knockout (arrowheads, J,L) and the presence of Cux1-positive neurons in deep layers (bracket, K).

Recent studies have demonstrated that several transcription factors and transcriptional regulators regulate the molecular identity of cortical neurons and ultimately laminar identity of pyramidal neurons [9,10]. In these cases, changes in laminar fate are altered and the axonal projections are correspondingly altered. Since a general change in laminar fate could explain reduction of callosal axons in Ngn2-/- embryos and the increase of laterally projecting axons after shRNA-mediated knockdown of Ngn2, we assessed whether there was a change in laminar fate in Ngn2-/- embryos or upon Ngn2 knockdown. We began by immunostaining Ngn2+/- and Ngn2-/- embryos at E18.5 for deep layer molecular markers (Figure 5A-F). Tbr1 is a transcription factor expressed primarily by layer 6 neurons [27], and CTIP2 is a transcription factor highly expressed in layer 5 neurons and weakly expressed in layer 6 neurons [28]. We found no expansion of Tbr1 or CTIP2 expression in Ngn2-/- cortices (Figure 5D-F) when compared to Ngn2+/- cortices (Figure 5A-C). Although we did not observe an expansion of deep layer neuronal fate, we wished to directly test whether superficial layer neurons were generated properly in the absence of Ngn2. To do this, we harvested Ngn2+/- and Ngn2-/- pups at P0, the last time point possible to assess Ngn2-/- mice. Here we immunostained for CTIP2 and Cux1, a transcription factor expressed in superficial layer neurons (layers 2 to 4) [29]. We found Cux1 expression in neurons located in layers 2 to 4, that is, just superficial to CTIP2-expressing neurons in both Ngn2+/- (Figure 5G-I) and Ngn2-/- cortices (Figure 5J-L). While we did not observe any change in expression of laminar markers between Ngn2+/- and Ngn2-/- cortices, we did observe the presence of CTIP2-expressing neuronal heterotopias ventral to layer 5 in the Ngn2-/- cortices (arrowheads in Figure 5J, L), and an increase of Cux1-positive neurons in layers V and VI (bracket in Figure 5K), illustrating the migration phenotype previously reported [15,16].

**Figure 6:**
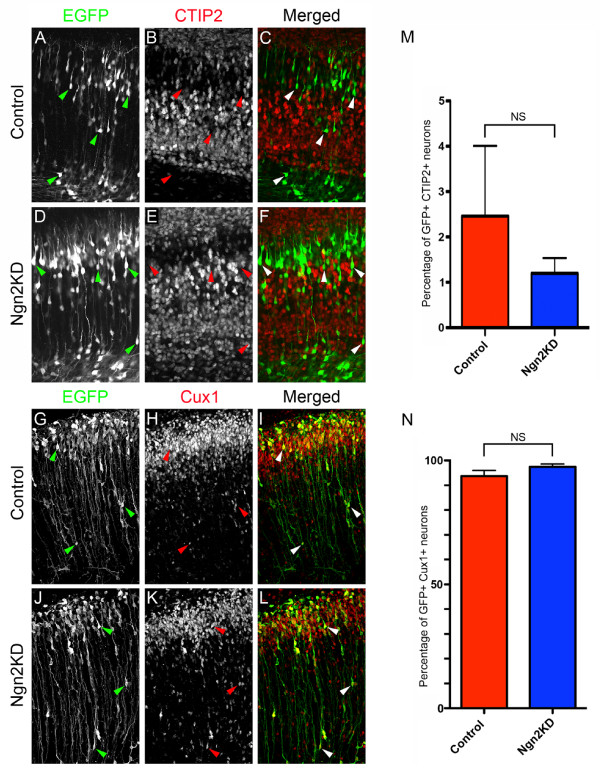
**Knockdown of Ngn2 expression does not induce a change in laminar fate**. E15.5 embryonic cortices were electroporated with plasmids encoding the YFP variant Venus and either control shRNA or shRNA targeting Ngn2. Following electroporation, organotypic slice cultures were prepared and cultured for 5 DIV. To identify any potential change in laminar fate, slices were stained for CTIP2, a molecular marker for deep layers (layers 5 to 6) and Cux1, a molecular marker for superficial layers (layers 2 to 4). **(A,D,G,J) **Electroporated cells were identified by immunostaining for EGFP. **(B,C,E,F) **CTIP2 immunostaining (B,E) and the merged images (C,F) demonstrated that very few electroporated neurons express the deep layer marker. **(M) **Quantification of these data shows no significant difference in the number of neurons that were immunostained for GFP and CTIP2. **(H-L) **Cux1 immunostaining (H,K) and merged images (I,L) show that most of the electroporated neurons express the superficial layer marker. **(N) **Quantification demonstrates that there is no significant difference in the number of neurons that were immunostained for GFP and Cux1. Interestingly, the location of the neurons within the cortex has no effect on the expression of laminar markers (arrowheads). NS, not significant. Error bars indicate standard deviation.

In addition to testing Ngn2-/- embryos for lamination defects, we also tested if there was a more subtle change in laminar fate that could be observed using shRNA-mediated knockdown of Ngn2. To do this we electroporated E15.5 cortices with control shRNA or shRNA targeting Ngn2. After 5 DIV, we performed immunofluorescent staining of electroporated slices with the laminar markers CTIP2 and Cux1. As expected, we found very few neurons containing the control shRNA that were CTIP2-positive (3.3%; Figure 6A-C, M). Just as we observed with the Ngn2-/- embryos, we found that shRNA-mediated knockdown of Ngn2 did not induce the expression of CTIP2 in superficial neurons containing the shRNA targeting Ngn2 (1.3%; Figure 6D-F, M). To confirm that the shRNA-mediated knockdown of Ngn2 did not alter the laminar fate of superficial neurons, we immunostained the cortices electroporated with either the control shRNA (Figure 6G-I) and the shRNA targeting Ngn2 with Cux1 (Figure 6J-L). We found that regardless of the shRNA electroporated, the vast majority of neurons expressed the layer II to IV marker Cux1 (control shRNA 94.8% and Ngn2KD 97.5%; Figure 6N). Based on the immunostaining of laminar markers in both Ngn2-/- embryos and slice cultures electroporated with shRNA targeting Ngn2, we found no altered laminar fate upon the loss of Ngn2. These results demonstrate that Ngn2 is required for the proper axonal projection of layer 2/3 neurons medially but not for the general specification of laminar fate.

### Superficial pyramidal neurons lacking Ngn2 project axons to many areas postnatally

**Figure 7:**
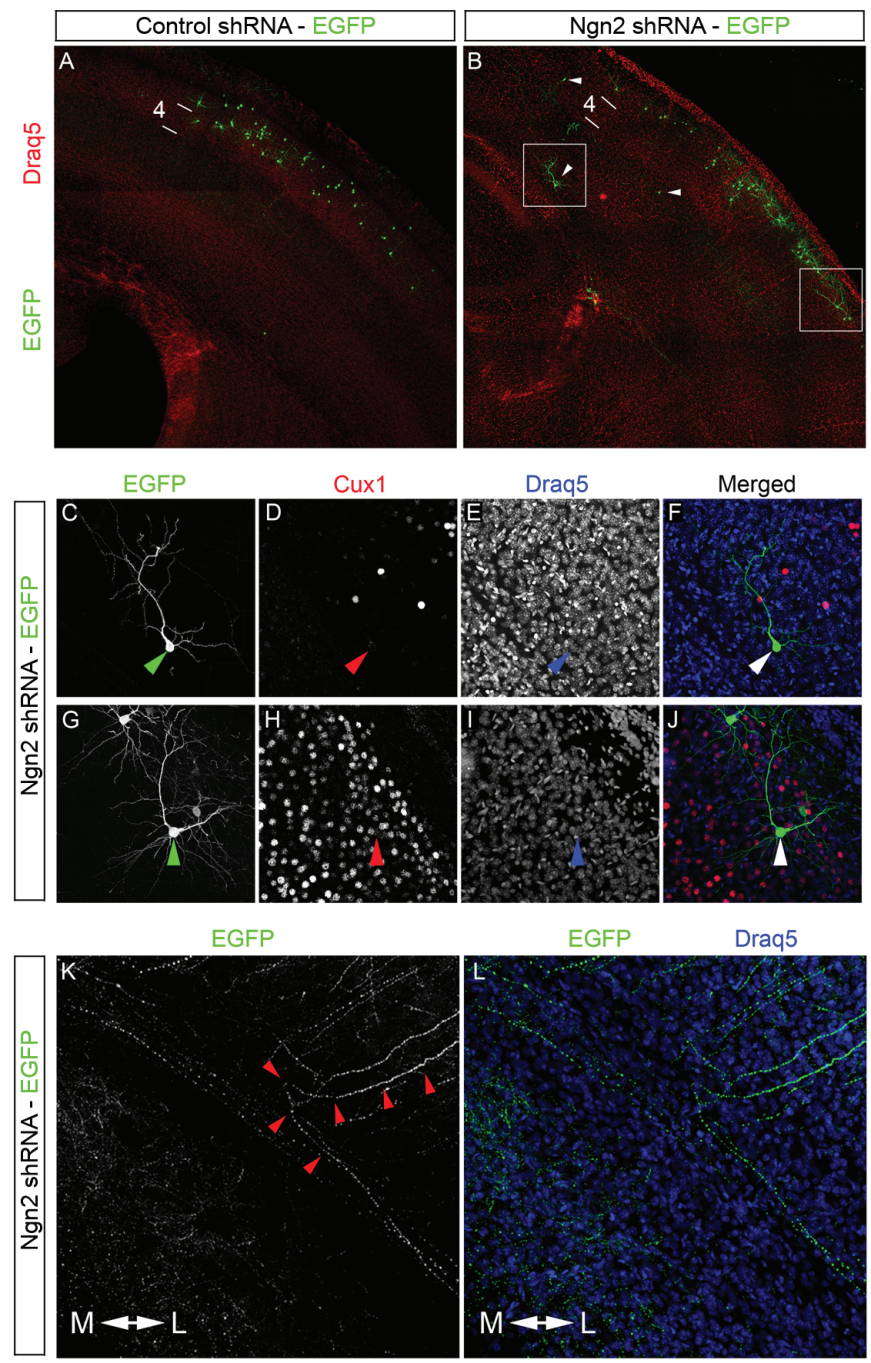
**Ngn2 knockdown by *in utero *electroporation reproduces previously described phenotypes attributed to Ngn2**. *In utero *electroporation of shRNA targeting Ngn2 reproduced several of the phenotypes we previously attributed to Ngn2. **(A,B) **Low magnification of the cortex at P14 electroporated with either the control shRNA (A) or the shRNA targeting Ngn2 (B) were immunostained for EGFP, and DNA was stained with Draq5. While no detectable phenotype was identified in cortices electroporated with the control shRNA (A), several phenotypes were identified in the cortices electroporated with the shRNA targeting Ngn2 (B). Arrowheads denote misplaced cells due to an inhibition of migration. Boxes identify neurons with either misoriented or multiple apical dendrites (shown in (C-J)). High magnification reveals the dedritic defects. **(C-J) **The neurons were immunostained with EGFP (C,G) to reveal morphology, Cux1 (D,H) to confirm laminar identity, and Draq 5 (E,I). (F,J) Merged images. **(K,L) ***In utero *electroporation also identified lateral projecting axons emerging from neurons located in the cortical plate.

Superficial neurons lacking Ngn2 have fewer callosally projecting axons (Figure 2) and acute knockdown of Ngn2 using shRNA induces superficial pyramidal neurons to project axons laterally (Figure 4). While the *ex vivo *cortical electroporation coupled with organotypic slice cultures allowed us to rapidly identify the errantly projecting axons upon Ngn2 knockdown, we were unable to decipher where these axons ultimately project postnatally. To assess where the misguided axons project, we used *in utero *electroporation of control shRNA and shRNA targeting Ngn2. The cortices of embryos were electroporated at E15.5 and the electroporated embryos were born and the mice were harvested at postnatal day 14 (P14) and immunostained for GFP to reveal the neuronal morphology and axonal projection, Cux1 to reveal laminar identity, and Draq5, a fluorescent nuclear stain. The long term *in utero *electroporation of shRNA targeting Ngn2 replicated the well-documented migration phenotypes attributed to Ngn2. Just as we have documented in the Ngn2-/- cortices (Figure 5J-L) [15], we found the presence of neurons located ventral to the upper layers of the cortex containing the shRNA targeting Ngn2 (Figure 7B), and we observed no inhibition in migration in neurons containing the control shRNA (Figure 7A). We do not believe that the inhibition of migration induced by the loss of Ngn2 is responsible for the axon guidance phenotype, since we see more erroneously projecting axons than misplaced cells. In addition, we found neurons located in the upper layers of the cortex with laterally projecting axons (Figures 4L and 7K, L). Just as we confirmed the impaired migration associated with a loss of Ngn2, we also replicated our previous results implicating Ngn2 in controlling dendritic morphology and orientation [15]. Upon shRNA-mediated knockdown of Ngn2, we found pyramidal neurons with apical dendrites improperly oriented (Figure 7C-F) and pyramidal neurons with multiple apical dendrites (Figure 7G-J).

**Figure 8:**
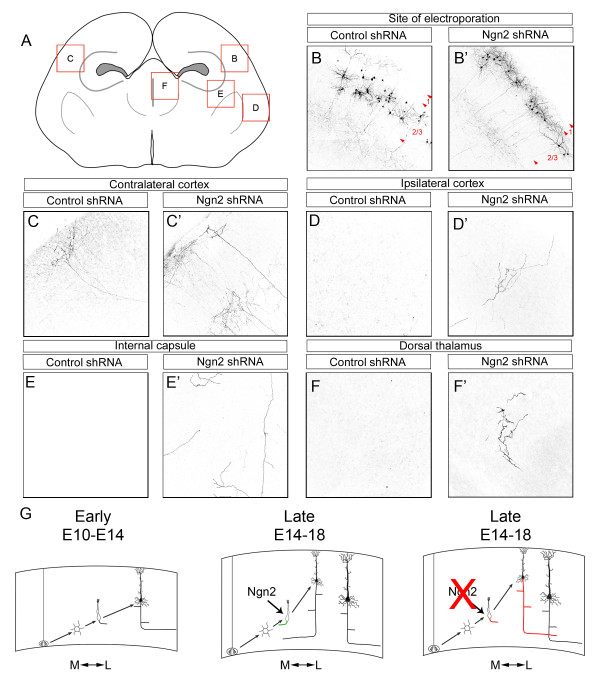
**The loss of Ngn2 results in cortical axons aberrantly projecting to cortical and subcortical targets *in vivo***. *In utero *electroporation of plasmids encoding the YFP variant Venus and either control shRNA or shRNA targeting Ngn2 were used to determine the final location of aberrantly projecting axons. Electroporated embryos were allowed to be born and survive to P14. Coronal sections were prepared and immunostained for GFP to enhance and reveal electroporated neurons and their axonal projections. **(A) **A diagram depicting the areas imaged. **(B-F') **Several areas were found to contain axons from neurons electroporated with control shRNA (B-F) and shRNA targeting Ngn2 (B'-F'). These areas include the zone of electroporation (B,B'), the contralateral cortex (C,C'), the ipsilateral cortex (D,D'), the internal capsule (E,E') and the thalamus (F,F'). Arrowheads indicate limits between layers. We found that several areas containing axons from neurons electroporated with the shRNA targeting Ngn2 (D',E',F') but not the control shRNA (D,E,F). **(G) **We have developed a model for the regulation of cortical axon guidance by Ngn2. At early stages (E10 to E14) when deep layer neurons (layers 5 to 6) are being generated, most of the axons initially project laterally. At later stages when superficial layer neurons (layers 2 to 4) are being generated, neurons differentially respond to cues within the cortex and project medially towards the corpus callosum. We believe Ngn2 is involved in this differential response. In the absence of Ngn2, there is a reduction in callosal axons and some of these axons project laterally towards cortical and subcortical targets.

To identify where aberrantly projecting callosal axons ultimately project, we imaged several regions in the brain (Figure 8A). We found that the vast majority of axons in cortices electroporated with the control shRNA (Figure 8B) innervated the contralateral cortex (Figure 8C). Rarely did axons from neurons containing the control shRNA project to other areas, such as the ipsilateral cortex (Figure 8D), internal capsule (Figure 8E), or thalamus (Figure 8F). The axons from neurons expressing shRNA targeting Ngn2 (Figure 8B') projected to several areas. Some axons from the neurons containing the shRNA targeting Ngn2 did successfully innervate the contralateral cortex (Figure 8C'). Interestingly, the misguided axons that initially projected laterally were found in many areas of the brain. We found an increase in axons present in the ipsilateral cortex (Figure 8D'). We also found many axons within the internal capsule (Figure 8E'), suggesting that some of the erroneously projecting axons were capable of exiting the cortex. Interestingly, we found that Ngn2KD neurons projected axons within the thalamus (Figure 8F'), proving that these axons were capable of innervating subcortical targets. The fact that acute knockdown of Ngn2 induces axons to erroneously project to areas both within and outside the cortex suggests that there is no default targeting of superficial neurons lacking Ngn2. These data support the notion that Ngn2 is important in regulating the initial axon guidance choice of callosal axons to project medially towards the corpus callosum, as erroneously projecting axons appear to innervate other brain regions randomly. Interestingly, we did not observe any defects in midline crossing of axons from neurons containing the shRNA targeting Ngn2. This would suggest that the defects in midline crossing and premature defasciculation of callosal axons may be cell non-autonomous. This is plausible as Ngn2 is expressed in the cingulate cortex and at the midline [30]. Based on these findings, we believe that Ngn2 is necessary for the guidance of callosal axons, and that an inhibition of migration is not responsible for the laterally projecting axons originating from superficial pyramidal neurons. Overall, the *in utero *electroporation of shRNA targeting Ngn2 allowed us to identify the location of erroneously projecting axons and further demonstrate its importance in the regulation of migration, dendritic morphology, and axonal projections during cortical development *in vivo*.

## Discussion

Here we demonstrate a novel role for Ngn2 during cortical development. We found that the genetic loss of Ngn2 results in a reduction of callosal axons and a malformation of the corpus callosum *in vivo*. When Ngn2 expression is knocked down acutely using shRNA in progenitors of superficial pyramidal neurons, many layer 2/3 neurons that normally project axons medially, now aberrantly project them laterally toward both cortical and subcortical brain regions. The change in axonal projection resulting from the loss of Ngn2 does not induce any dramatic change in laminar fate, at least with regard to the expression of transcriptional regulators such as CTIP2 or Cux1, although a small proportion of neurons permanently fail to reach their final position in layer 2/3 (Figures 5 and 8). Taken together our results show that Ngn2 coordinates the acquisition of many of the cardinal features of pyramidal neurons in the developing cortex, including neurotransmitter expression [13], migration properties and dendritic morphology [15], and axonal projections (the present study and [25]). Previous results [14] showed that expression of layer-specific markers of layers 5/6, such as Tbr1 and ER81, were upregulated in the Ngn2-/- compared to wild-type mice but that markers of layer 2/3, such as Cux1, were unchanged. Our results confirm that Ngn2 does not seem to have a major role in determining the transcriptional identity of superficial layer 2/3 neurons (Figures 4 and 5) but rather have a significant effect on the specification of their axon projections (see below).

The first interpretation of our results is that during the second half of neurogenesis (that is, after E14), when neurogenesis switches from producing subcortically projecting layer 5/6 neurons to producing mostly cortico-cortical and contralateral/midline projecting neurons (layer 2-4), Ngn2 plays an instructive function in intermediate progenitors by directly or indirectly specifying midline projection 'fate' (Figure 8G). The second alternative interpretation of our results is that the Ngn2 function during the second half of cortical neurogenesis is to repress the lateral projection fate during the production of superficial layer 2/3 neurons, thereby allowing neurons to acquire a 'cortical projection fate' [14] (Figure 8G). Future experiments will test these two mechanisms by identifying the transcriptional mechanisms (transactivation or repression) underlying their function in the cortex.

Since Ngn2 is expressed throughout neurogenesis (present results, but also see [13-15,19], this suggests that the transcriptional function of Ngn2 changes over time. During early stages of cortical neurogenesis (such as E12.5), Ngn2 is primarily playing a proneural function due to high expression in dividing progenitors [18,19], but at later time points Ngn2 has additional roles in the acquisition of the phenotypic traits associated with pyramidal neurons, including neurotransmitter expression [13,14], migration [15,16] and axon guidance (present study and [25]). This could be explained by the subtle change in expression pattern over time and/or by post-translational modifications affecting Ngn2's ability to partner with various transcriptional regulators [15,17,31]. Our data support this as we found Ngn2 expression in the upper SVZ and the IZ, and most of these cells also expressed Tbr2 (Figure 1E-H), a marker for IPCs at the later time point of E16.5, when superficial layer neurons are differentiating. Since the Ngn2 expression pattern changes as the cortex develops, we hypothesize that Ngn2 is capable of inducing differential gene expression as the cortex develops due to the differential expression of transcriptional co-activators and differences in accessibility of transcriptional targets due to epigenetic regulation by chromatin-modifying proteins. Future studies will be needed to identify which of the known transcriptional targets of Ngn2 [32-34] or novel transcriptional targets of Ngn2 regulate the switch in axonal projections over time, and to test how Ngn2 differentially regulates these gene(s) during cortical development.

Our data suggest that the initiation of the axon is a directed process leading to the guidance of the axon medially within the intermediate zone. We found this initial projection often occurred in immature neurons prior to forming a leading process and before initiating migration (Figure 1I). This raises an interesting question of whether direct transcriptional targets of Ngn2 regulate the initial projection of the superficial pyramidal neuron or if downstream transcription factors are responsible for the observed phenotype. Several transcription factors are directly and indirectly downstream of Ngn2, including transcription factors expressed in the SVZ (Tbr2 and NeuroD4) [32,35], in the IZ (NeuroD1) [14,15], and in the cortical plate (NeuroD2 and MEF2C) [14,33]. Recently, a downstream target of Ngn2, the small GTPase Rnd2, was found to play an important role in the control of radial migration of pyramidal neurons [16]. Furthermore, Rnd2 was sufficient to rescue the inhibition of migration in Ngn2-/- embryos. Interestingly, Rnd2 is a direct target of both Ngn2 and NeuroD1 [16], suggesting that Ngn2 is capable of directly and indirectly regulating the transcription of a gene necessary for migration. Identifying the gene(s) that are directly responsible for the directed axon guidance of sub-cortical versus medially projecting neurons will be of great interest. Several studies have investigated genes downstream of Ngn2. Not surprisingly, receptors for several axon guidance ligands, including Netrins, Slits, Semaphorins, and Ephrins, are down-regulated in Ngn2-/- embryos [14,33,34], and some of these receptors were found to be direct targets of Ngn2, while others are presumably indirect transcriptional targets. Therefore, we believe that Ngn2 is likely regulating, both directly and indirectly, the gene expression underlying the initial projection of callosal axons medially. Future experiments will need to test which Ngn2 downstream targets participate in the guidance of layer 2/3 axons towards the midline.

## Conclusions

We, along with others, have identified that Ngn2 is crucial for the proper formation of cortical circuitry. While Ngn2 was first identified as a proneural transcription factor, further studies have demonstrated that Ngn2 regulates many of the defining features of pyramidal neurons. Elegant genetic studies previously showed that Ngn2, along with Ngn1, a close homolog presenting an overlapping expression pattern, specify the neurotransmitter fate of pyramidal neurons [14]. In addition, Ngn1 is largely sufficient to compensate for the proneural deficit associated with the loss of Ngn2, and likely to compensate for some other phenotypes associated with Ngn2. This may explain the partial penetrance of our phenotypes. In addition to the expression of glutamate as a neurotransmitter and the repression of ventral telencephalic fate through repression of Mash1 expression, Ngn2 is required for the proper location of pyramidal neurons and acquisition of pyramidal dendritic morphology. Neuronal morphology and laminar position are both crucial to the formation of proper neural circuits. The present study demonstrates that Ngn2 regulates how pyramidal neurons innervate target areas by regulating the first axon guidance decision made by layer 2/3 pyramidal neurons to project laterally towards the midline, which underlies the formation of cortical circuits. Further studies of Ngn2 transcriptional targets will lead to a better understanding of the molecular mechanisms underlying its function during cortical circuit formation.

## Abbreviations

BDA: biotinylated dextran amine; DIV: days *in vitro*; E: embryonic day; EGFP: enhanced GFP; GFP: green fluorescent protein; IPC: intermediate progenitor cell; IZ: intermediate zone; Ngn2: Neurogenin2; NPC: neural progenitor cell; PBS: phosphate-buffered saline; shRNA: short hairpin RNA; SVZ: subventricular zone; VZ: ventricular zone; YFP: yellow fluorescent protein.

## Competing interests

The authors declare that they have no competing interests.

## Authors' contributions

RH designed and performed the experiments and co-wrote the manuscript. FP designed and supervised the experiments and co-wrote the manuscript. Both authors read and approved the final manuscript.
